# Simulation study on hydrogen concentration distribution in hydrogen blended natural gas transportation pipeline

**DOI:** 10.1371/journal.pone.0314453

**Published:** 2024-12-03

**Authors:** Weiqing Xu, Yongwei An, Shuangjie Yan, Rui Li, Maolin Cai, Guanwei Jia

**Affiliations:** 1 School of Automation Science and Electrical Engineering, Beihang University, Beijing, China; 2 Pneumatic and Thermodynamic Energy Storage and Supply Beijing Key Laboratory, Beijing, China; 3 School of Physics and Electronics, Henan University, Kaifeng, China; 4 General Institute of Science and Technology of National Petroleum and Natural Gas Pipeline Network Group Co.,Ltd, Langfang, Hebei, China; Southwest Petroleum University, CHINA

## Abstract

Hydrogen is a clean energy source, and blending it with natural gas in existing pipeline networks is a key transition solution for transportation cost reduction. However, during the transportation process, a non-uniform distribution of hydrogen concentration occurs in the pipeline due to gravity. Therefore, it is necessary to study the hydrogen concentration distribution law of hydrogen-blended natural gas in pipelines. The undulation and ball valve pipelines, which are common in transport pipelines, were constructed in this study. The effects of the undulation angle, height, pipeline diameter, ball valve opening, and temperature on the distribution of the hydrogen concentration were investigated using computational fluid dynamic (CFD) methods. The results indicated that the hydrogen concentration gradient changed gently with the larger diameter of the undulating pipeline, minimizing hydrogen accumulation. Higher undulation angle and smaller height differences reduces the hydrogen accumulation risk. Increasing vertical height difference of the pipeline from 5 m to 15 m increased the hydrogen volume fraction gradient by1.3 times. In the ball valve pipeline, the velocity fluctuation decreased as the ball valve opening increased. However, the hydrogen accumulation phenomenon was obvious. The opening increased from 25% to 100% and the hydrogen volume fraction gradient increased more than two times. Selecting delivery conditions with low hydrogen blending ratios, high temperatures, low pressures, and high flow rates reduces the occurrence of hydrogen buildup in the pipeline.

## 1. Introduction

The continuous increase in global greenhouse gas emissions has caused frequent extreme weather occurrences. The promotion of energy transformation and structural upgrading has gradually reached a global consensus [1]. Hydrogen is a secondary energy source with great potential for development [2, 3]. It has a high calorific value, various energy storage and conversion methods, stable energy output, etc. [4]. It is an important carrier to help realize the goal of "peak carbon and carbon neutrality " and build a clean, low-carbon [5], safe, and efficient energy system [6]. The International Hydrogen Energy Association (IHEA) predicts that hydrogen energy will account for 18% of global end-use energy consumption in 2050 [7, 8]. Hydrogen production by electrolyzing water using renewable energy is an effective method of power abandonment and energy conversion [9, 10]. The conversion efficiency of hydrogen production from electrolyzed water reaches up to 72.47% [11], and the average cost is $2.66-$3.54/kg H_2_ [12]. However, because hydrogen production is concentrated in the Midwest of China in provinces and regions that are rich in fossil or renewable energy sources, hydrogen use is concentrated in the southeast coast of China. The geographical mismatch between hydrogen-producing and hydrogen-using locations necessitates long-distance transportation. Commonly used hydrogen transportation methods include tube trailer, liquid hydrogen tankers, and manifold bundles [13]. However, these methods have a low transport capacity and high transportation costs, accounting for approximately one-third of the final selling price [14]. However, these methods are only suitable for short-distance transportation. High transportation costs pose challenges in the development of hydrogen energy [15]. Pipeline transportation allows high flow rates, long distances, and high efficiency, thereby significantly reducing transportation costs. The total cost of transporting an average of 1 ton of hydrogen for 1 km is approximately $1 to $10 [16]. However, pure hydrogen pipelines are expensive, at approximately 1 million dollars per mile [17], which is three times more than that of natural gas pipelines. Pure hydrogen pipelines are less frequently laid, with a total mileage of only 400 km, which is only 0.36% of natural gas pipelines [18]. Most hydrogen transmission pipelines are located in chemical parks, and in-service pipelines are approximately 100 km long. Thus, only localized hydrogen delivery can be achieved. The length of natural gas pipelines in service in China is as high as 118,000 km. The existing natural gas trunk pipeline and branch pipeline networks are extensive. Injected hydrogen into natural gas pipelines at a specific ratio for blended transportation is the optimal approach for reducing the cost of hydrogen [19]. At the same time, it makes full use of the energy storage capacity of the natural gas pipeline network and realizes large-scale storage and delivery of hydrogen. As China is the world’s largest importer of liquefied natural gas (LNG), its degree of foreign dependence is high [20]. Hydrogen blending into natural gas pipelines to replace some fuels can alleviate the pressure on the natural gas supply and promote energy structure transformation [21]. The demand for hydrogen transportation will gradually increase as the amount of hydrogen produced and used increases. The increase in the ratio of hydrogen blending is helpful to increase the transportation efficiency and the amount of hydrogen transported. However, an increase in the hydrogen blending ratio will change the characteristics of the gas being transported in the pipeline. The composition of the gas mixture affects the efficiency and safety of the natural gas transport system. Currently, research and projects on hydrogen-blended natural gas use blending ratios of less than 20%. For example, the “Liaoning Chaoyang Gas Hydrogen Blending Demonstration Project” has a 10% hydrogen blend and has been operating safely for more than one year. The “HyP Gladstone” and “HyP Murry Valley” projects in Australia injected hydrogen into the natural gas network at a blend of 10% [22]. USA’s “HyBlend” project plans to blend hydrogen at a rate of 1–30% and gradually increase the hydrogen blend [23, 24]. The USA’s “SoCalGas” project plans to ultimately achieve 20% hydrogen blending [25]. I. A. Gondal [26] studied the effect of hydrogen blending on natural gas infrastructure and gas quality. The permissible limit of the hydrogen blending ratio was obtained as 10%. Studying the hydrogen concentration distribution of hydrogen-blended natural gas with up to 30% hydrogen blending ratio in the pipeline can not only improve the amount of delivered hydrogen, but also provide the necessary reference and experience for future theoretical research and demonstration projects of large-scale hydrogen-blended natural gas delivery.

Natural gas pipeline transportation technology is currently mature. Because of the high diffusivity of hydrogen, its density is 1/8 of that of natural gas, and its leakage rate is more than three times that of natural gas. During transportation, gas friction increases the resistance to flow of the gas, leading to an uneven distribution of the gas flow in the pipeline. The formation of vortices and shear layers may be triggered, which can further affect the distribution of hydrogen. The diffusion coefficient of hydrogen is much higher than that of other components in natural gas, and hydrogen diffuses relatively quickly in natural gas. During transportation, the intensity of turbulence of hydrogen-blended natural gas in the pipeline is high, and the effect of molecular diffusion on hydrogen concentration stratification is relatively small. In regions with larger concentration gradients, hydrogen will diffuse more rapidly, thus helping to minimize hydrogen accumulation. This introduces new technical difficulties and safety risks in the transportation of hydrogen-blended natural gas [27], as the physical and chemical properties of hydrogen differ from those of natural gas. When hydrogen is blended with natural gas, hydrogen may accumulate in the structure of the transportation pipeline, thereby affecting the safety of pipeline transportation. During pipeline transportation, as the gas flows, the hydrogen-blended natural gas shifts from the initial blended uniform state owing to the combined effects of gravity and molecular motion [28]. Hydrogen, with its lower relative molecular mass, tend to move towards the top of the pipeline, whereas methane, with its higher relative molecular mass, moves towards the bottom of the pipeline. Hydrogen accumulates at the top of the pipeline and methane accumulates at the bottom [29]. The ball valve’s long-term operation in a high concentration of hydrogen can cause pipeline wear and fatigue due to hydrogen-induced stress. And it affects the equipment of the whole hydrogen-blended natural gas transport system [30], such as compressors [31], pipelines [32], welds [33], valves [34], and storage [35]. Hydrogen accumulation in a pipeline can affect its use and even increase the risk of explosion [36]. It has been suggested that gas diffusion is a relatively slow process [37]. Gas accumulation may result only from an initial inhomogeneous mixing state [38]. S. Peng et al. [39] concluded that hydrogen-blended natural gas in a closed region does not ultimately produce a large accumulation of hydrogen, but the stratification is not obvious when the height difference is very small, but favors hydrogen stratification when the height difference is large and the temperature is low. On the other hand, S. Peng et al. are compensating for the limitation of very small altitude in the molecular dynamic model calculations by increasing the gravity field, which is at odds with the actual situation. While, A. Marangon, et al. [40] observed the inhomogeneity of the gas mixture concentration under both confined and ventilated conditions. The results show that the gas mixture is vertically stratified at high flow rates and large temperature differences. S. Ren, et al. [41] analyzed the effect of the pipeline diameter and hydrogen blending ratio on the hydrogen accumulation of hydrogen-blended natural gas. The results showed that the smaller the pipeline diameter and the larger the hydrogen blending ratio, the more prone the pipeline was to hydrogen accumulation. C. Liu, et al. [42] concluded that hydrogen accumulation occurred in low-flow rate pipelines. Gas mixture accumulation tends to occur at lower velocities, higher pressures, lower temperatures, and higher hydrogen mixing volume fractions. Y. N. Shebeko, et al. [43] found experimentally that in hydrogen-methane mixtures in closed containers there is a buildup of gaseous hydrogen about 4 hours after stopping the filling. The hydrogen volume fraction at the top and bottom of the vessel differed by 10%. The above studies have shown the existence of gas accumulation and stratification phenomena in mixtures of hydrogen and natural gas. However, the conditions of hydrogen-blended natural gas during transportation are complicated and there is a certain gap with the experimental conditions. It is also necessary to fully analyze the influence of model structure, external conditions, operating conditions and other factors on the hydrogen concentration distribution of hydrogen-blended natural gas. The hydrogen concentration distribution in the flow of a pipeline has been studied less extensively. In real pipeline networks, undulating fluctuations of pipelines are a common phenomenon. Such fluctuations may have an impact on the flow state and concentration distribution of the fluid in the pipeline. For hydrogen-blended natural gas pipeline transportation, the difference in physical and chemical properties between hydrogen and natural gas may lead to an uneven distribution of hydrogen concentration at the pipeline undulation fluctuations. Although upward accumulation of hydrogen will occur in the actual pipeline network, it is not certain that a high concentration accumulation zone can be fully formed. When the gases are blended, there will be more intense molecular movement within them, which will promote uniform blending to a certain extent. However, due to the large difference in density between hydrogen and natural gas, the distribution trend caused by the density difference is larger than the uniform trend caused by molecular motion, and the phenomenon of concentration stratification occurs on a macro level. The phenomenon of non-uniform distribution of concentration occurs in undulating pipelines due to gravity. At the same time, during the flow process of blended hydrogen natural gas pipeline, the phenomenon of the hydrogen component shifting to the top of the pipeline may also occur in the case of small overall flow rate due to the tendency of hydrogen to drift to the top of the pipeline. This uneven distribution may bring a series of problems, such as hydrogen embrittlement, increased risk of hydrogen leakage, and reduction of pipeline transportation efficiency. Therefore, it is necessary to study the hydrogen concentration distribution in hydrogen-blended natural gas transmission pipelines. Exploring the changing laws and influencing factors of hydrogen concentration distribution. The current research status of hydrogen-blended natural gas stratification is first analyzed in this paper; then the physical model, mathematical model and boundary conditions in the simulation process are described in detail; after that, the effects of the pipeline undulation angle, height and pipeline diameter changes on the hydrogen aggregation in the pipeline are analyzed; and finally, the effects of the opening of the ball valve, the hydrogen-blended natural gas volume ratio, the pressure, the velocity and the temperature changes on the hydrogen aggregation in the pipeline are analyzed. The results of this study have important engineering significance for the safe transportation of hydrogen-blended natural gas transportation systems.

## 2. Modeling of transportation pipeline

### 2.1 Pipeline modeling

**Fig 1:**
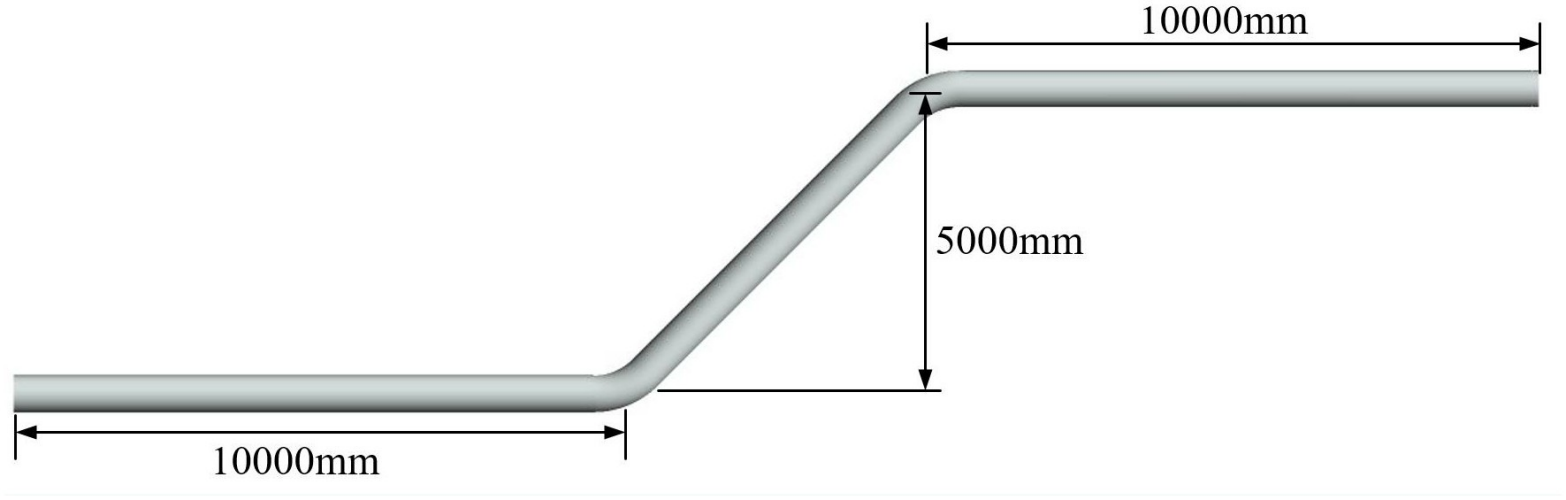
Undulation pipeline geometric modeling.

Long-distance transportation pipelines often need to pass through areas with undulating terrain. Undulating pipelines, which can be elevated over obstacles, are a common type of transmission pipelines, as shown in Fig 1. The pipeline model was modeled using the Geometry module in Fluent module. In order to improve the computational efficiency and save the computational resources, the pipeline parameters refer to the diameter of the pipeline of the fourth line of the West-East Natural Gas Pipeline, and the pipeline model is reduced by 2:1. The inner diameter of the pipeline was set as 600 mm. The left side shows the input of the uniformly blended hydrogen-blended natural gas, and the right side shows the outlet. The turning radius of the rising pipeline was taken as 2 times the diameter of the pipeline (1,200 mm) to reduce pressure loss. The rise height of the pipeline was 5,000 mm, the rise angle was 45°, and the horizontal pipelines were all 10,000 mm. The rise height was 5,000 mm, and the rise angle was 45°.

**Fig 2:**
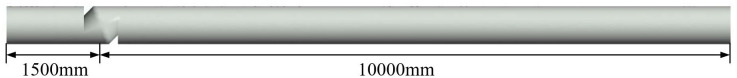
Ball valve pipeline geometric modeling.

A ball valve pipeline was constructed to control the pressure and flow rate of a long-distance pipeline, as shown in Fig 2. The initial opening of the ball valve line was 50% and the ball valve rotates by 45°. The diameter of the ball valve was 900 mm, and the center point of the valve was 1500 mm from the inlet and 10000 mm from the outlet to reduce backflow interference.

### 2.2 Mathematical modeling

The flow of hydrogen-blended natural gas through a pipeline satisfies three basic principles of fluids: the equation of conservation of mass, the equation of conservation of momentum, and the equation of conservation of energy. Natural gas and hydrogen follow the equation of conservation of mass during blending and flowing through the pipeline [44]:

$$\frac{\partial \rho }{\partial t}+\frac{\partial \left(\rho \nu_{x}\right)}{\partial x}+\frac{\partial \left(\rho \nu_{y}\right)}{\partial y}+\frac{\partial \left(\rho v_{z}\right)}{\partial z}=0$$ (1)

where *v*_*x*_, *v*_*y*_, and *v*_*z*_ are the components of velocity in *x*, *y*, and *z* directions, respectively, in m/s; *ρ* is the density of the fluid with the unit of kg/m^3^.

Conservation of momentum equation in blending and flow [45]:

$$\begin{array}{l} \frac{\partial (\rho v_{x})}{\partial t}+div(\rho v_{x}{\bar{v}}_{x})=div(Ugradv_{x})-\frac{\partial p}{\partial x}+S_{x}\\ \frac{\partial (\rho v_{y})}{\partial t}+div(\rho v_{y}{\bar{v}}_{y})=div(Ugradv_{y})-\frac{\partial p}{\partial y}+S_{y}\\ \frac{\partial (\rho v_{z})}{\partial t}+div(\rho v_{z}{\bar{v}}_{z})=div(Ugradv_{z})-\frac{\partial p}{\partial z}+S_{z} \end{array}$$ (2)

where ${\bar{v}}_{x}$,${\bar{v}}_{y}$, and ${\bar{v}}_{z}$ are the fluid velocity vectors in m/s; *U* is the gas blending uniformity; *p* is the local relative pressure relative to the working pressure in Pa; *S*_*x*_, *S*_*y*_, and *S*_*z*_ are the generalized source terms for momentum conservation; div is the dispersion; and grad is the gradient.

The law of energy conservation in the blending process and flow [46]:

$$\frac{\partial (\rho T)}{\partial \text{t}}+\frac{\partial \left(\rho v_{x}T\right)}{\partial x}+\frac{\partial \left(\rho v_{y}T\right)}{\partial y}+\frac{\partial \left(\rho v_{z}T\right)}{\partial z}=\frac{\partial }{\partial x}\left(\frac{k}{c_{p}}\frac{\partial T}{\partial x}\right)+\frac{\partial }{\partial y}\left(\frac{k}{c_{p}}\frac{\partial T}{\partial y}\right)+\frac{\partial }{\partial z}\left(\frac{k}{c_{p}}\frac{\partial T}{\partial z}\right)+{\text{S}}_{T}$$ (3)

where *k* is the fluid heat transfer coefficient; *c*_*p*_ is the specific heat capacity in J/(kg·K); *T* is the temperature in K; *S*_*T*_ is the heat source within the fluid and the part of the fluid’s mechanical energy converted to thermal energy due to viscous effects.

The most applicable standard *κ-ε* model (*κ* is the turbulent kinetic energy; *ε* is the dissipation rate) is chosen for gas blending calculations. Calculation accuracy was ensured while reducing the calculation volume [47].

The *κ* equation:

$$\rho \left(u\cdot \nabla \right)\kappa =\nabla \cdot \left[\left(\mu +\mu_{\tau }\sigma_{x}\right)\nabla \kappa \right]+P_{x}-\beta_{0}\rho \omega \kappa$$ (4)

where *μ* is the kinetic viscosity coefficient; *μ*_*T*_ is the turbulent viscosity coefficient in Pa·s; *u* is the gas velocity vector in m/s; *σ*_*κ*_ is the *Pr* corresponding to the turbulent kinetic energy; *Pr* is the Prandtl number; *P*_*κ*_ is the term generated due to the turbulent kinetic energy due to the velocity gradient; and *β*_*0*_ is a constant.

The *ε* equation:

$$\rho \left(u\cdot \nabla \right)\varepsilon =\nabla \cdot \left[\left(\mu +\mu_{\tau }\sigma_{z}\right)\nabla \varepsilon \right]+\tau \frac{\varepsilon }{\kappa }P_{x}-\rho \beta_{0}\varepsilon^{2}$$ (5)

where *ε* is the kinetic energy dissipation rate; *σ*_*ε*_ is the *Pr* corresponding to the kinetic energy dissipation rate; and *τ* is a constant.

With other boundary conditions unchanged, different turbulence models (standard *κ-ε* model, RNG *κ-ε* model, standard *κ*-*ω* model, and LES model) are examined for their influence on the simulation results. For a hydrogen molar fraction of 10%, a ball valve structure due to 25% opening and 1 MPa pressure is selected. To calculate the difference between the average hydrogen concentration at the outlet and the theoretical calculation results for different turbulence models. Table 1 shows the simulation results for different turbulence models. It can be seen that using different turbulence models, the errors in the simulated values of the hydrogen blending ratio at the outlet location are less than 1%. When the standard *κ-ε* model is used, the average hydrogen blending ratio at the outlet of the pipeline is 9.987%, which is 0.13% different from the theoretical value and has the smallest error. Therefore, the standard *k-ε* model is selected as the final turbulence model in this paper.

**Table 1:** Simulation results for different turbulence models.

Turbulence model	Simulated value of outlet H_2_ molar fraction	Theoretical value of outlet H_2_ mole fraction	Errors (%)
Standard *κ-ε* model	0.09987	0.1	0.13%
RNG *κ-ε* model	0.09969		0.31%
Standard *κ*-*ω* model	0.09986		0.14%
LES model	0.09977		0.23%

### 2.3 Meshing and independence verification

The mesh density affects the accuracy of the machine’s calculation of the flow field state of hydrogen-blended natural gas. Typically, the higher the mesh density, the higher the accuracy of the simulation results. However, additional computational resources and longer computational times were required. Mesh-independent verification ensures the accuracy of the calculation results and saves computational resources and time. For the mesh quality, the closer the mesh skewness is to 0, the better the mesh is, and the mesh skewness of 0~0.25 indicates excellent mesh quality. The cell quality and orthogonal quality of the mesh are closer to 1, the more perfect the mesh is. The average cell quality and orthogonal quality of the mesh with excellent quality are not less than 0.7. Due to the complex structure of the ball valve, the model under the condition of 25% opening, 1 MPa pressure, and 10% hydrogen blending ratio is selected to verify the mesh irrelevance of the ball valve pipeline. The results are summarized in Table 2. The mesh was divided into three sizes, and the number of mesh cells were 128855, 221277, and 433107, respectively. The quality of the mesh is more than 0.75, the orthogonal quality of the mesh is around 0.79, and the skewness is less than 0.2. The quality of the divided mesh reaches the excellent level. The increase in the mesh density resulted in a smaller change in the hydrogen volume fraction at the outlet; therefore, the effect of the mesh density on the simulation results can be ruled out. Therefore, a medium number of meshes was selected for subsequent simulation analysis.

**Table 2:** Mesh independence verification.

Mesh Type	Number of modules	Number of nodes	Average skewness	Average cell quality	Orthogonal mass	Mole fraction of H_2_ at the outlet
Sparse	128855	25386	0.14555	0.7726	0.79208	10.001
Medium	221277	42410	0.1264	0.7882	0.79234	10.001
Dense	433107	81089	0.10378	0.79713	0.79387	10.0008

### 2.4 Boundary condition setting

The direction of gravity was vertically downward with a magnitude of 9.81 m/s^2^. A pressure solver was selected. Methane and hydrogen gases were then added to the material properties. The outlet pressure was set as 1 MPa. The inlet is a velocity inlet with an inlet velocity of 2.5 m/s. The inlet and outlet temperatures were set to 298 K. The turbulence model is the standard *κ*-*ε* model. The wall surface was a stationary, no-slip, adiabatic wall. Standard initialization is performed before simulation. The pipeline is set to be filled with hydrogen-blended natural gas with a hydrogen blending ratio of 10% at initialization. For hydrogen blending ratios of 20% and 30%, the pipeline is initialized to be filled with hydrogen-blended natural gas with a hydrogen blending ratio of 20% and 30%, respectively. A residual threshold of 10^−6^ was set for all physical quantities, and the calculation was considered to have converged when the residuals of all physical quantities were reduced below the set threshold of 10^−6^.

## 3. Analysis of hydrogen concentration distribution in undulating pipelines

Seven cases were set up to analyze the hydrogen accumulation patterns of hydrogen-blended natural gas in undulating pipelines. The simulation results for the seven model structures are listed in Table 3. The effects of the undulation angle, height, and pipeline diameter on the hydrogen accumulation were analyzed. Gravity was the main factor influencing gas accumulation. The gas concentrations in the horizontal pipeline diameters were identical. Therefore, only the distribution of gas concentration in the direction of gravity at the outlet was considered.

**Table 3:** Parameters of undulation pipeline and simulation results.

No.	Angle (°)	Height (m)	Pipeline diameter (mm)	Maximum hydrogen volume fraction (%)	Minimum hydrogen volume fraction (%)	Hydrogen volume fraction gradient (%)
1	30	5	600	10.0183	9.9865	0.0319
2	45	5	600	10.0152	9.9913	0.0238
3	60	5	600	10.0152	9.9926	0.0225
4	45	10	600	10.0153	9.9886	0.0267
5	45	15	600	10.0187	9.9875	0.0312
6	45	5	400	10.0159	9.9850	0.0309
7	45	5	800	10.0149	9.9924	0.0225

**Fig 3:**
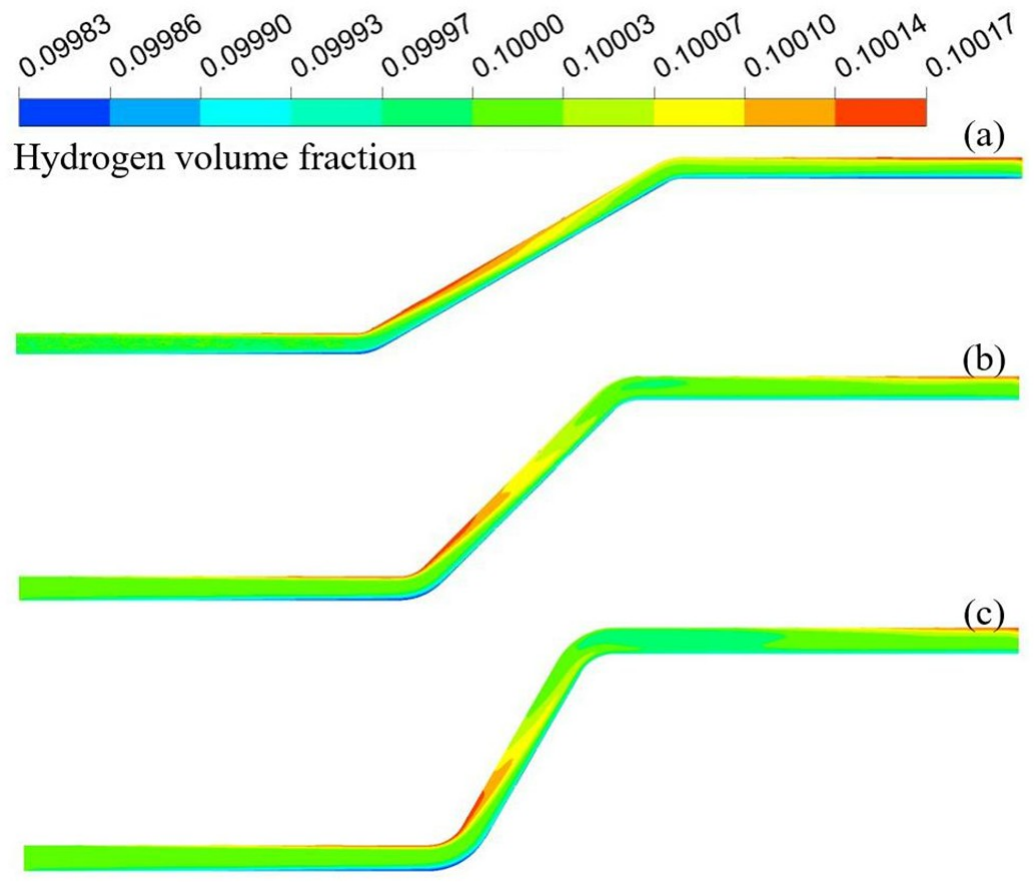
Hydrogen volume fraction distribution at different undulation angles (a) 30°; (b) 45°; (c) 60°.

**Fig 4:**
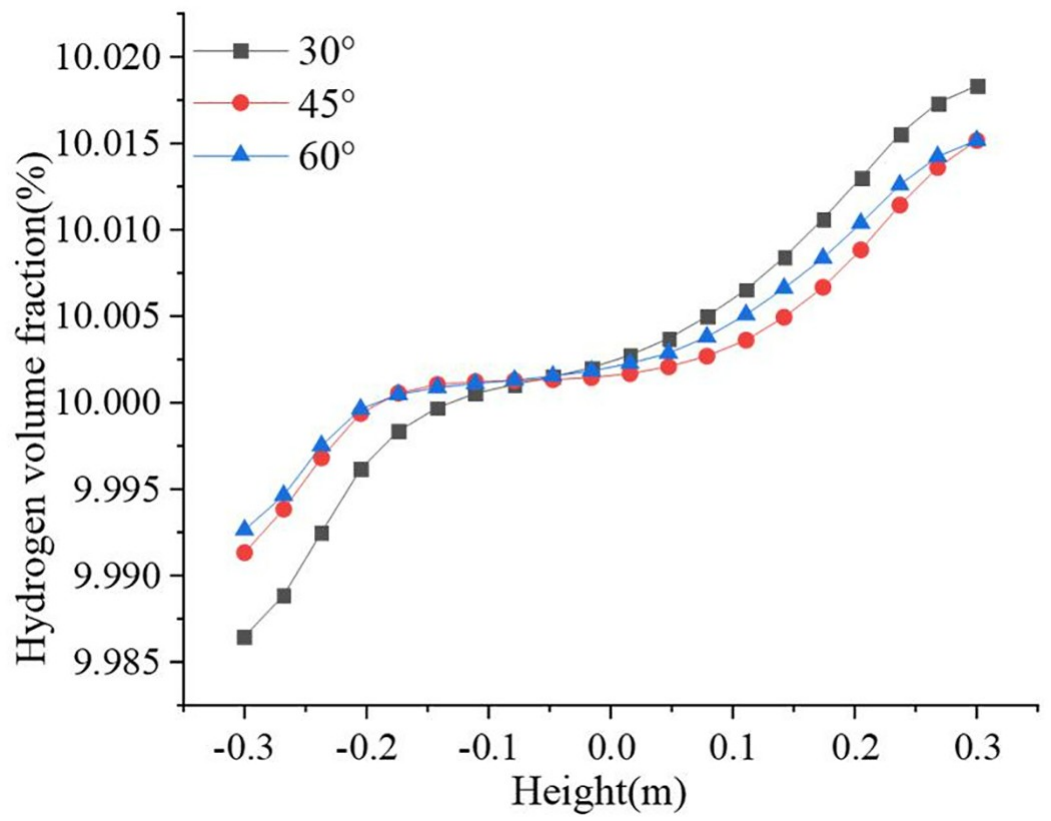
Variation of hydrogen volume fraction at different undulation angles.

Fig 3 shows the hydrogen volume fraction distributions at different undulation angles. Fig 4 shows the variation in the hydrogen volume fraction along the pipeline diameter. The distribution trends of hydrogen were similar for the three sets of models. Hydrogen accumulated at the end of the blend fluid at the bottom. Hydrogen flowed along the pipeline wall in the upward section, and hydrogen accumulation disappeared near the top. However, after a certain degree of stabilization, hydrogen accumulation appeared again at the top end. The smaller the undulation angle, the smaller the impact on the right wall when the fluid rises, and the smaller the reverse impact. The maximum velocities in the pipeline at undulation angles of 30°, 45°, and 60° were 3.51 m/s, 3.52 m/s, and 3.57 m/s, respectively. The more stable the flow rate of the gas in the ascending pipeline, the faster the accumulation occurs when it reaches the top. The maximum hydrogen volume fraction occurred at an ascent angle of 30°, reaching 10.0183% with the hydrogen volume fraction gradient reaching at maximum of 0.0319%. The hydrogen volume gradient gradually decreases with the rise angle. The hydrogen volume gradient decreases to 0.0225% at a rise angle of 60°. Compared with the 30° hydrogen volume fraction gradient decreased by 30%. Therefore, hydrogen accumulation can be suppressed by increasing the angle of the pipeline, fluid disturbance, and turbulence energy.

**Fig 5:**
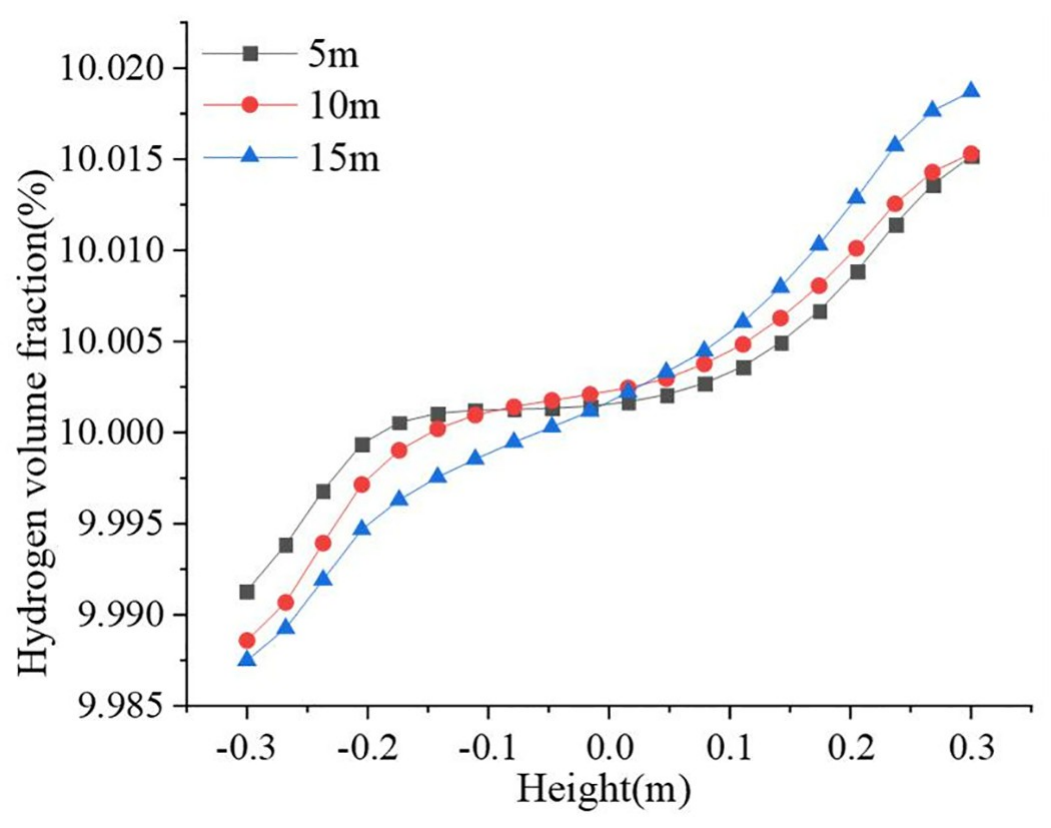
Hydrogen volume fraction at different height differences.

Fig 5 shows the hydrogen volume fraction change curves at different height differences. With an increase in the height difference, the gradient of the hydrogen volume fraction in the pipeline gradually increased. The hydrogen aggregation phenomenon became more and more obvious, and the gradient reached up to 0.0312%. The hydrogen volume fraction gradient widens by a factor of 1.3 when the height of the pipeline was increased from 5 to 15 m. Gravity significantly influences the difference in hydrogen concentration along the pipeline diameter. Therefore, for natural gas risers and transmission pipelines with large height differences, the focus should be on observing the hydrogen accumulation. If the hydrogen concentration exceeds a critical value, effective measures must be taken in time to avoid accidents.

**Fig 6:**
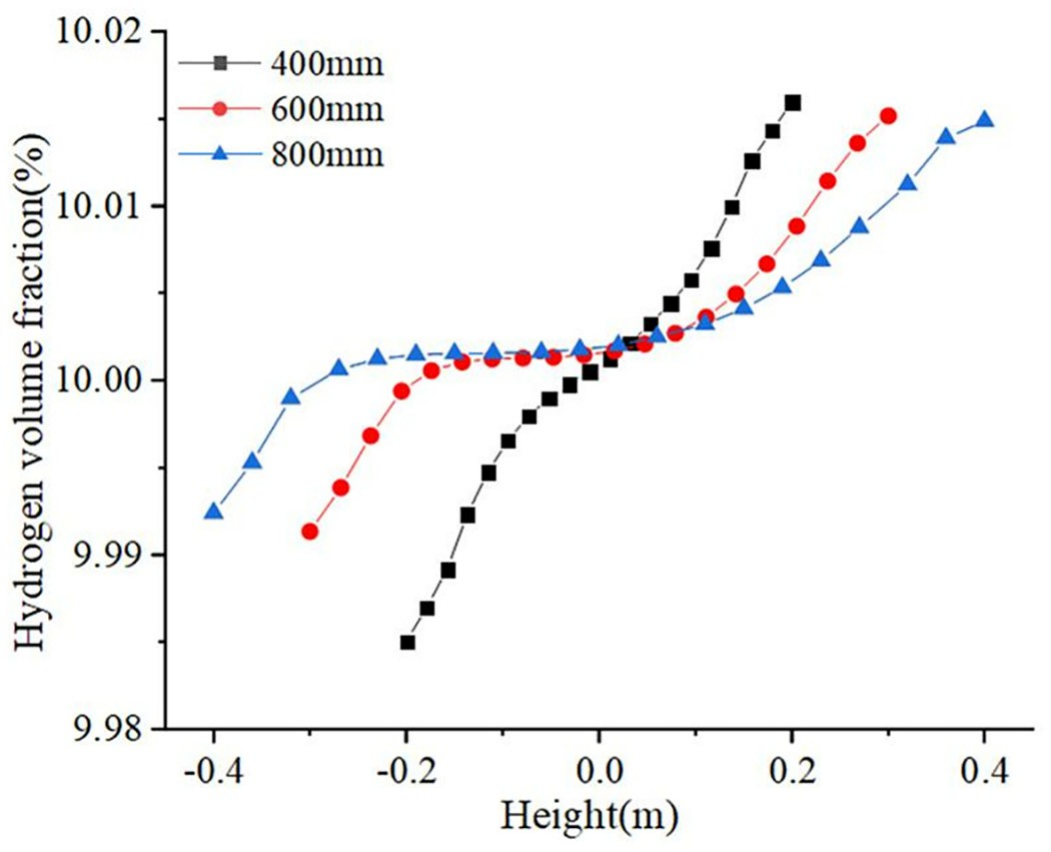
Variation of hydrogen volume fraction for different pipeline diameters.

Three pipeline diameters–400 mm, 600 mm, and 800 mm—were set up for modeling. The variation in hydrogen volume fraction in the pipeline is shown in Fig 6. With an increase in the pipeline diameter, the gradient of the hydrogen volume fraction gradually decreased, and the hydrogen accumulation phenomenon weakened. When the pipeline diameter was 400 mm, the hydrogen volume fraction gradient was the largest, reaching 0.0309%. The hydrogen volume fractions at the top and middle of the pipeline were relatively close to each other. Owing to the different pipeline diameters, the larger the pipeline diameter, the smoother the change in the gradient when moving the same distance up or down from the center. The larger the pipeline diameter, the less pronounced the hydrogen accumulation in the pipeline. Therefore, in the selection of pipeline diameters, large sizes of pipeline diameter should be chosen. A smoother gradient change was obtained, which reduced hydrogen accumulation. The use of larger diameter pipelines requires, on the one hand, more materials and more sophisticated construction techniques, which will increase the cost of construction and maintenance. On the other hand, an increase in pipeline diameter may require more land and resources to lay and maintain these pipelines, which will also increase costs. While increasing the diameter of the pipeline may reduce the risk of hydrogen build-up, a cost-benefit trade-off needs to be made with a combination of considerations including safety, cost, efficiency, and sustainability. Too large a pipe diameter may result in to slow a flow of hydrogen, increasing the probability of hydrogen buildup and the risk of leakage. Too small a pipe diameter may result in too fast a hydrogen flow rate, increasing friction losses and hydrodynamic shocks, thus increasing the risk to the system. In practice, a detailed assessment and analysis is required to determine the most appropriate pipe diameter and delivery option based on the specific situation.

## 4. Analysis hydrogen distribution of ball valve pipelines

**Fig 7:**
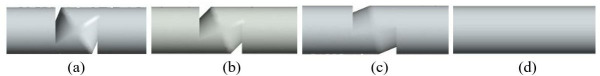
Localized schematic of ball valves with different openings (a) 25%; (b) 50%; (c) 75%; (d) 100%.

Valves have the functions of cutting off, regulating flow, decentralizing flow, etc., and play an important role in the transmission and distribution pipeline network. In the transmission process of hydrogen-blended natural gas, there is a need for the frequent use of valves to constantly adjust the flow and pressure to meet different geographical requirements. Ball valve structure is simple, requiring only a 90° rotation to realize full open and full closed flow. Its operation is simple, convenient, and easy to maintain and repair. A pipeline with a ball valve was used in the simulation. Considering the different throttling conditions encountered in practical applications, four ball valve openings of 25%, 50%, 75%, and 100% are selected. A localized schematic of the ball valve pipeline with different openings is shown in Fig 7. Valve pipeline models of 12 different structures were set up. The effects of the ball valve opening, hydrogen blending volume ratio, pressure, velocity, and temperature on hydrogen aggregation were analyzed separately, and the results are listed in Table 4.

**Table 4:** Valve parameters and simulation results.

No.	Opening (%)	HBR (%)	P (MPa)	Velocity (m/s)	T (K)	Maximum hydrogen volume fraction (%)	Minimum hydrogen volume fraction (%)	Hydrogen volume fraction gradient (%)
1	25	10	1	2.5	273	10.0053	9.9955	0.0098
2	50	10	1	2.5	273	10.006	9.9946	0.0118
3	75	10	1	2.5	273	10.0067	9.9941	0.0126
4	100	10	1	2.5	273	10.0107	9.9894	0.0213
5	50	20	1	2.5	273	20.0102	19.9915	0.0187
6	50	30	1	2.5	273	30.0117	29.9903	0.0215
7	50	10	1	2.5	298	10.0058	9.9950	0.0108
8	50	10	1	2.5	323	10.0055	9.9954	0.0100
9	50	30	3	2.5	273	10.0175	9.9847	0.0327
10	50	30	5	2.5	273	10.0288	9.9755	0.0533
11	50	10	1	5	273	10.0029	9.9970	0.0059
12	50	10	1	7.5	273	10.0019	9.9978	0.0041

**Fig 8:**
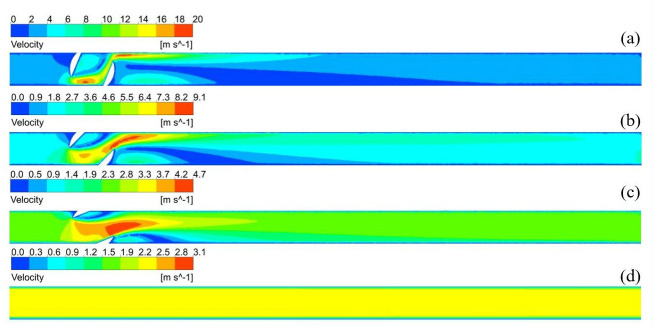
Velocity distribution at different openings (a) 25%; (b) 50%; (c) 75%; (d) 100%.

The gas velocity changes considerably at the valve, as shown in Fig 8. The smaller the opening, the more drastic the change in the effective flow cross-sectional area. When the valve was not fully opened, the throttling effect of the ball valve resulted in changes in the cross-sectional area of the flow, making the fluid flow state more complex. When the gas flowed into the ball valve, due to a sudden reduction in the effective flow cross-sectional area, it prompted the first acceleration, converting pressure energy into kinetic energy. When the gas flowed out of the ball valve, the effective cross-sectional area of the flow suddenly increased, and the fluid expanded. The expansion of the fluid promoted a second acceleration. The expansion of the fluid promoted a second acceleration. At 25% of the valve opening, the velocity change was the largest, reaching up to 20.1 m/s. At 50% and 75% of the valve openings, the maximum velocity were 9.1 m/s and 4.6 m/s, respectively. The opening of a ball valve directly determines its flow capacity. When the ball valve opening is small, the flow capacity of hydrogen is limited and the flow rate is slowed down, which may lead to hydrogen accumulation near the valve or in the pipeline. When the opening of the ball valve is larger, the flow capacity of hydrogen through the valve is increased and the flow rate is accelerated, which helps to reduce the accumulation of hydrogen in the pipeline. With an increase in distance, the flow rate gradually stabilized, and the velocity was stable at 2.5 m/s. The smaller the opening, the longer the distance required for fluid stabilization. The distances required for velocity stabilization are 7 m, 5 m, and 2 m for 25%, 50%, and 75% openings, respectively. When the valve is 100% open, it can be considered as a straight pipeline, the overall velocity does not change. The hydrogen-blended natural gas was maintained at a velocity of 2.5 m/s flowing through the pipeline.

**Fig 9:**
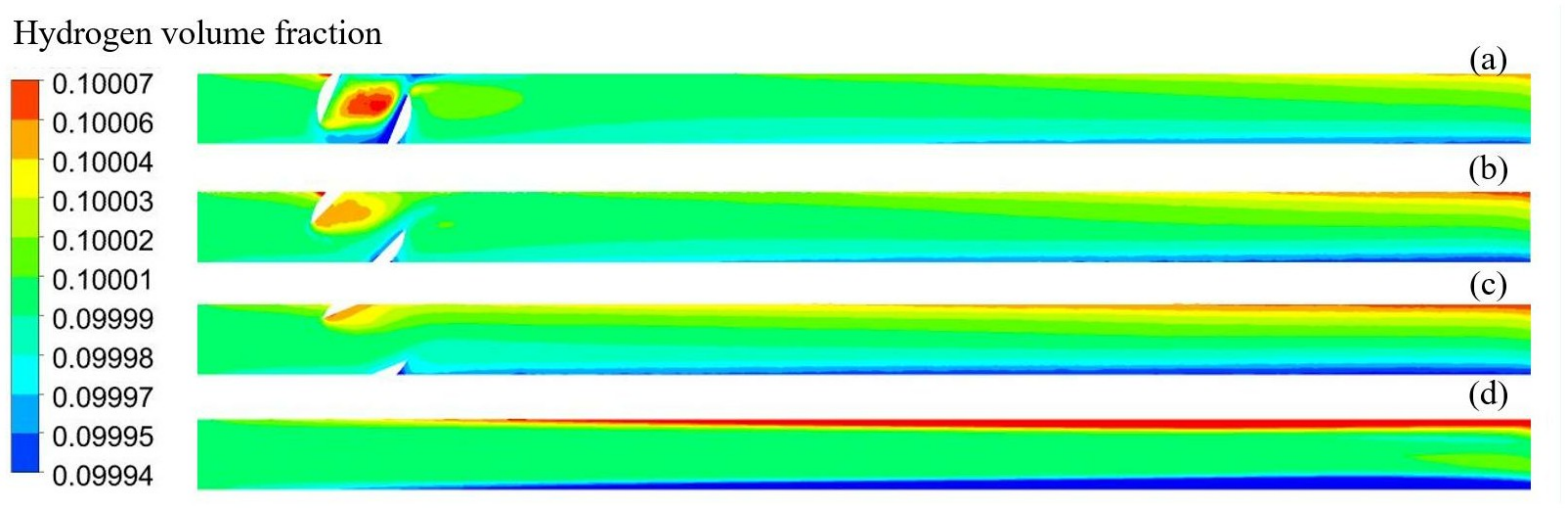
Distribution of hydrogen volume fraction at different openings (a) 25%; (b) 50%; (c) 75%; (d) 100%.

**Fig 10:**
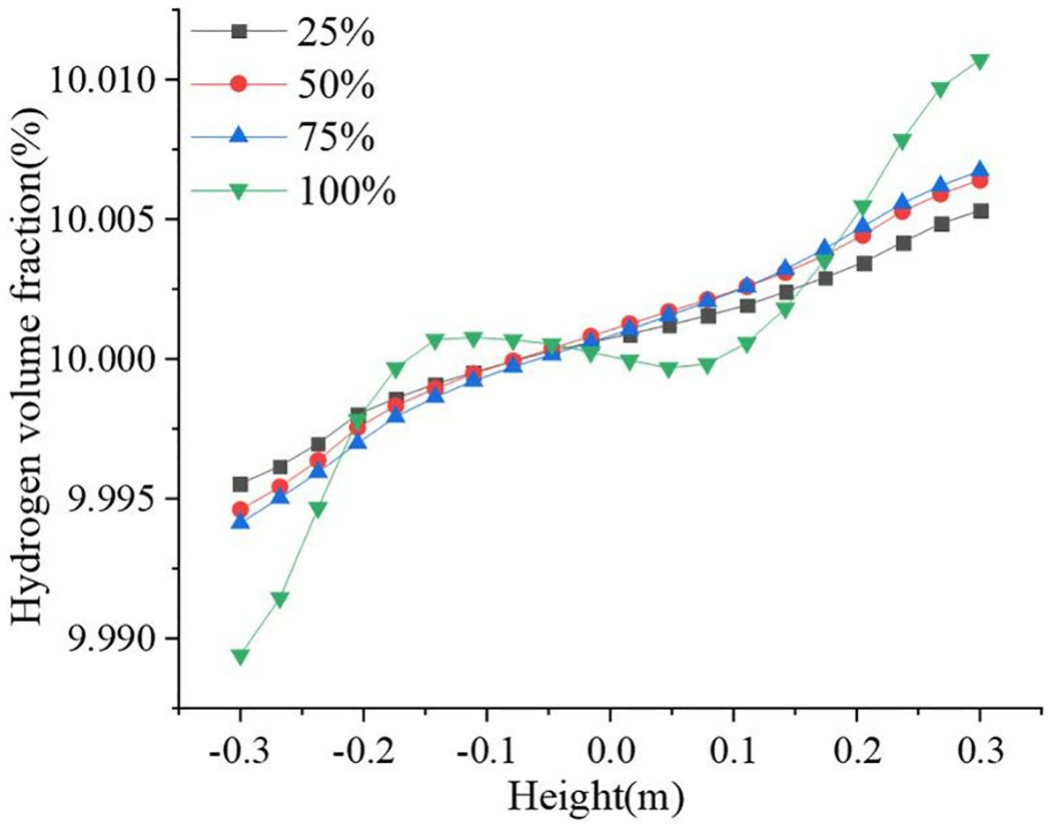
Variation of hydrogen volume fraction at different openings.

Fig 9 shows the distribution of the hydrogen volume fraction at different openings; hydrogen accumulation was observed at all four openings. Fig 10 shows the variation in the hydrogen volume fraction in the direction of the pipeline diameter at different openings. The hydrogen concentration changes in the direction of gravity. The gradient of the hydrogen volume fraction in the direction of gravity gradually increased with the valve opening. The smaller the opening, the larger the velocity change, and the larger the velocity, the larger the perturbation, making the fluid less likely to accumulate. For 25%, 50%, 75%, and 100% openings, the locations of hydrogen accumulation at the outlet were 1 m, 3 m, 5 m, and 8 m. The smaller the opening, the more backward and less obvious the appearance of hydrogen accumulation. At 100% opening, the flow field structure in the pipeline is similar to that of a straight pipeline, and the turbulence intensity in the flow of hydrogen-blended natural gas is minimized, and the maximum turbulence intensity in the pipeline is 13.5%. When the valve is not fully opened, the hydrogen-blended natural gas flow through the valve will be disturbed by the valve structure, and the turbulence intensity of the gas mixture increases. 25%, 50%, and 75% opening, the maximum turbulence intensity in the pipeline is 82.6%, 50.4%, and 39.6%, respectively. So, at 100% valve opening, the turbulence intensity inside the pipeline is weaker and the hydrogen is more prone to stratification. As the flow distance increases, the hydrogen concentration is more prone to stratification and the hydrogen volume fraction profile increases steeply. Combined with Fig 8 analysis, owing to the first three openings appearing as fluid acceleration and the upper left part of the ball valve being a low-velocity region, hydrogen moves up to gather in the upper left part. When the ball valve is not fully open, the pipeline and ball valve structures have dead space, and hydrogen aggregation occurs easily. The flow rate in the pipeline was lower when it was 100% open, so the hydrogen concentration accumulation was the most obvious phenomenon.

**Fig 11:**
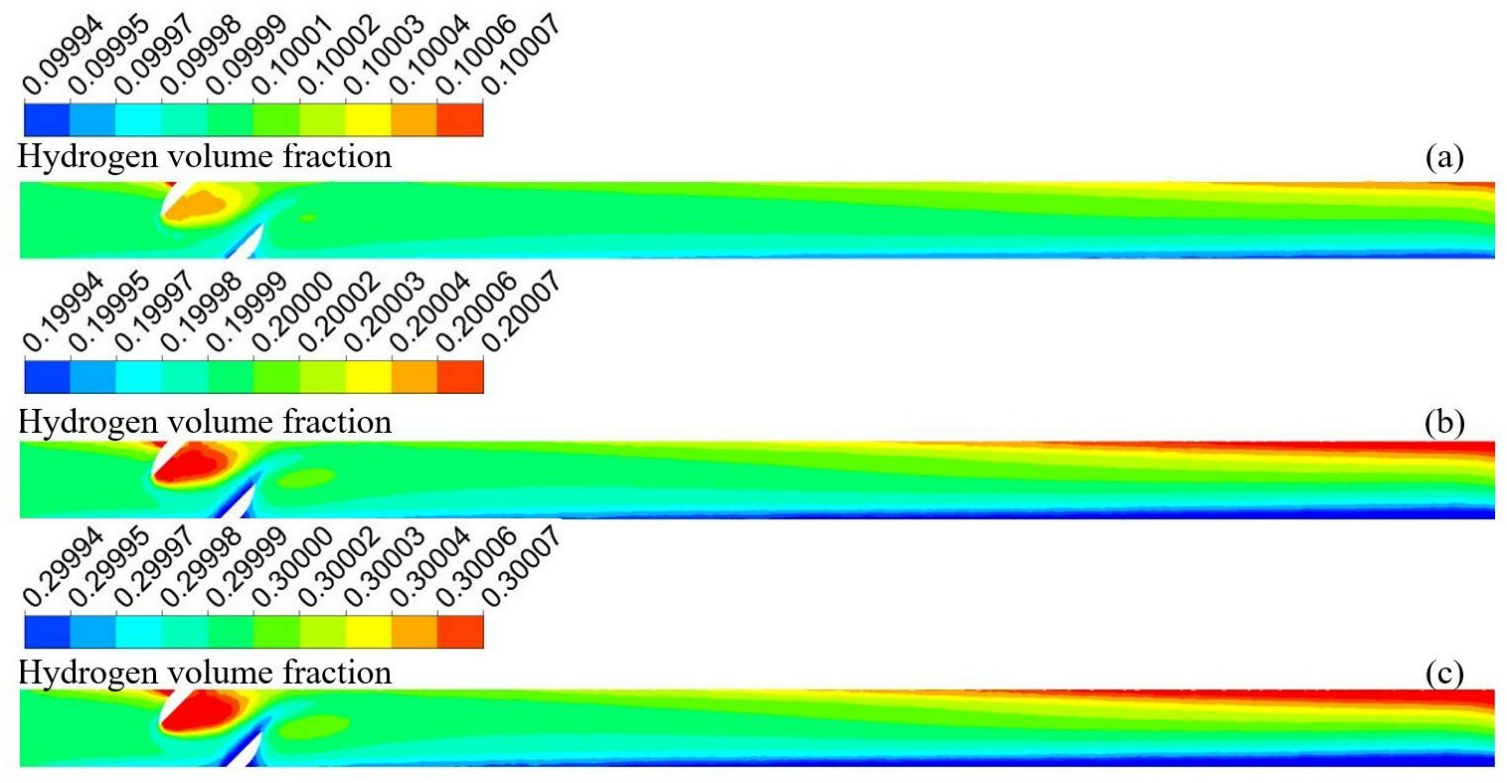
Distribution of hydrogen volume fraction at different hydrogen blending ratios (a) 10%; (b) 20%; (c) 30%.

**Fig 12:**
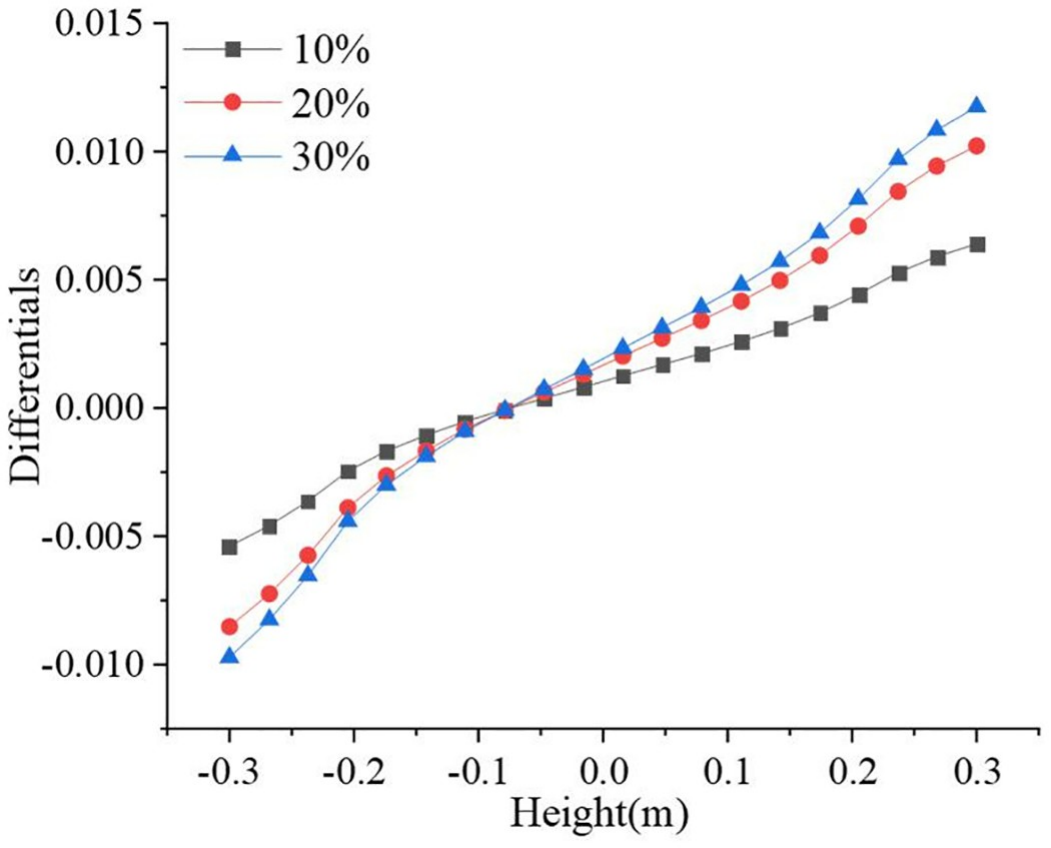
Hydrogen volume fraction change in the pipeline.

The valve opening is not always maintained at 100% during actual pipeline transportation. The operating conditions of hydrogen-blended natural gas pipelines are more complicated. The preset valve opening was 50%, and it is also necessary to analyze the effects of other changes in operating parameters on hydrogen accumulation. These parameters include the hydrogen blending ratio, pressure, velocity, and temperature. The inlet hydrogen blending ratios were set to 10%, 20%, and 30%, respectively. Due to the substantial difference in hydrogen concentration between the different hydrogen blending ratios, a benchmark value was introduced for comparison. The baseline values for hydrogen concentration in the pipeline are 10%, 20% and 30% for hydrogen blending ratios of 10%, 20% and 30%, respectively. Fig 11 shows the distributions of the hydrogen volume fractions at the three hydrogen blending ratios. Fig 12 shows the differences between the different hydrogen blending ratios with respect to the benchmark value. The volume fraction of hydrogen, which has a small relative molecular weight along the direction of gravity, gradually increased with increasing height, and the overall trend was consistent. Increasing the hydrogen blending ratio significantly increased the hydrogen content of the pipeline. The hydrogen accumulation phenomenon was more obvious. For 10%, 20%, and 30% hydrogen blending volume ratios, the hydrogen accumulation occurs at 8 m, 6 m, and 5 m from the valve, respectively. The 10% hydrogen blending volume ratio showed the smoothest change in hydrogen volume fraction and the smallest gradient of hydrogen volume fraction. As the hydrogen blending ratio increased, the hydrogen volume fraction gradient also increased. It increased from 0.0118% to 0.0215%, expanding 1.8 times. The maximum hydrogen volume fraction at this time is 30.0117%.

**Fig 13:**
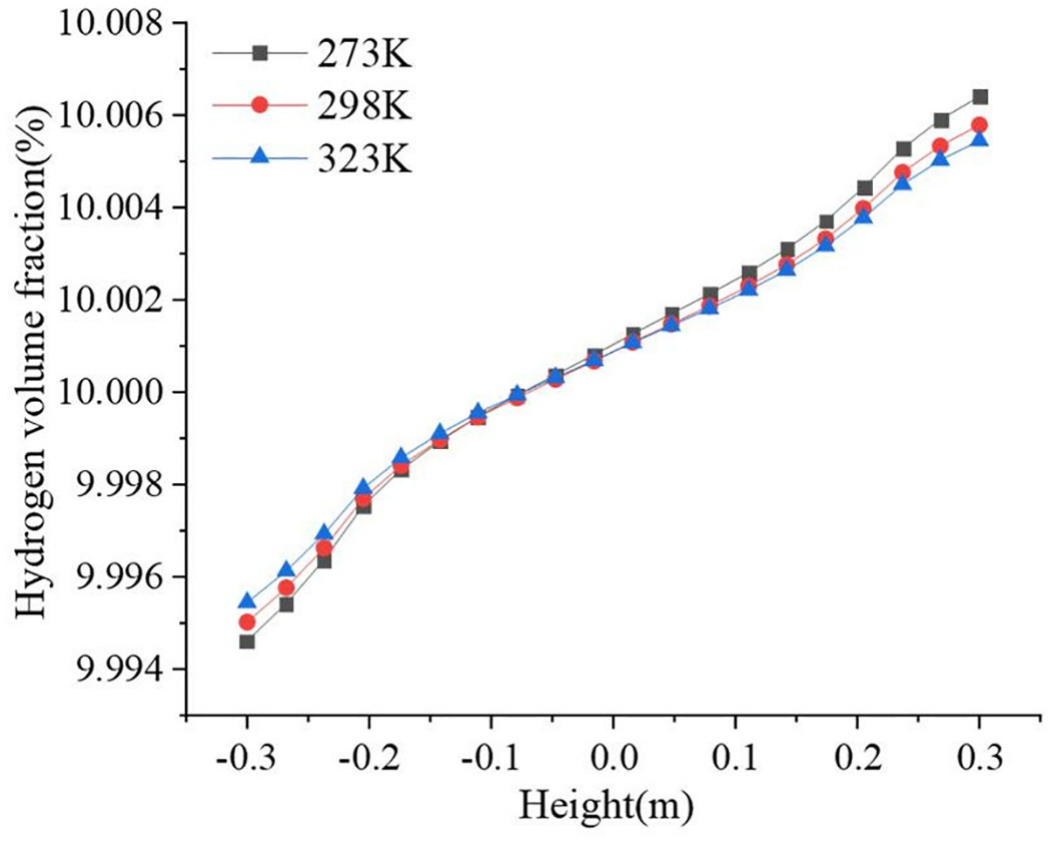
Variation of hydrogen volume fraction in the pipeline at different temperatures.

The inlet temperatures were set to 273 K, 298 K, and 323 K. The variations in the hydrogen volume fraction in the pipeline at the three inlet temperatures are shown in Fig 13. The hydrogen volume fraction along the direction of gravity increased progressively with increasing altitude. Higher temperatures have higher molecular internal energies and violent molecular motion, which can better resist hydrogen accumulation caused by gravity. The gradient of hydrogen volume fraction is 0.0118%, 0.0108%, and 0.0100% at 273 K, 298 K, and 323 K, respectively. As the inlet temperature increases, the hydrogen volume fraction gradient in the pipeline diameter direction decreases. The hydrogen concentration decreased and the minimum hydrogen volume fraction was 9.9946%. Therefore, to achieve safe transport in hydrogen-blended natural gas pipelines, it is important to increase the temperature during the transport process as much as possible. However, higher temperatures are not always better. The possible negative effects of such high temperature environments on the pipeline system must be considered at the same time, in particular thermal stresses and material degradation. Thermal stresses may lead to deformation, cracking or even rupture of the pipeline, thus affecting the safety and reliability of the hydrogen transport system. Prolonged thermal stress can accelerate fatigue damage and shorten the service life of pipelines. Material degradation reduces the strength and toughness of pipelines and increases the risk of leakage and rupture. Ensure the safety and reliability of hydrogen delivery systems in high-temperature environments through reasonable design, material selection and maintenance measures.

**Fig 14:**
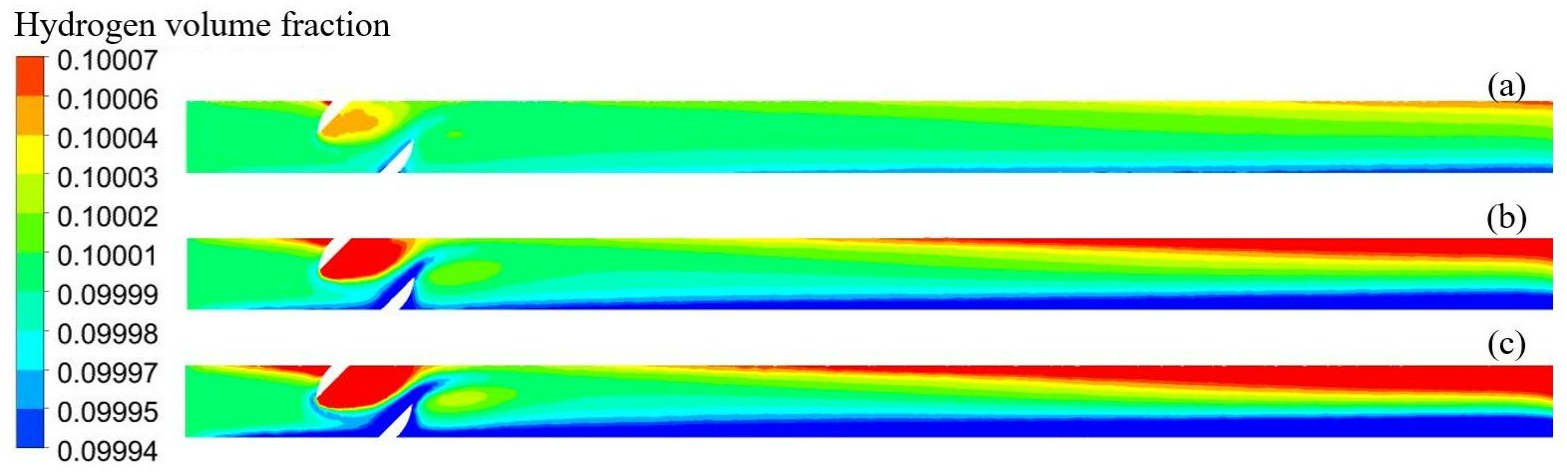
Hydrogen volume fraction distribution at different pressures (a) 1 MPa; (b) 3 MPa; (c) 5 MPa.

**Fig 15:**
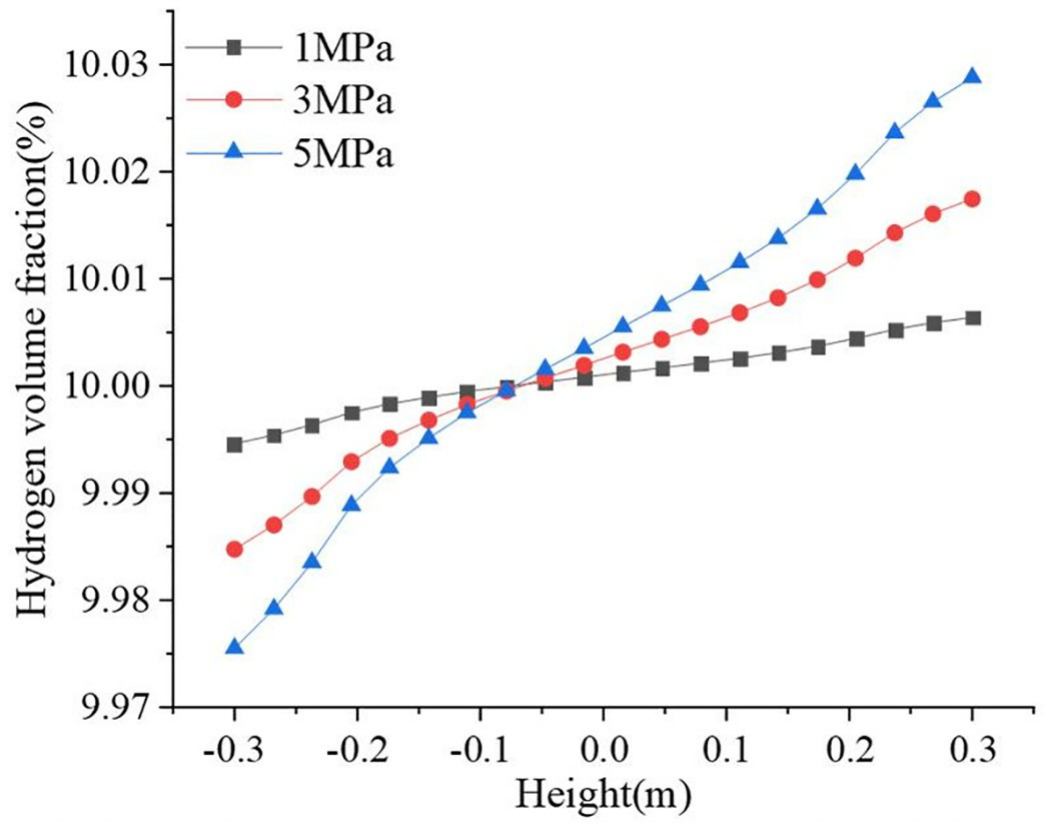
Variation of hydrogen volume fraction along the direction of the pipeline.

Hydrogen accumulation became more pronounced with increasing pressure. The hydrogen volume fraction along the pipeline diameter gradually becomes larger with the increase in height, and the maximum hydrogen volume fraction was 10.0288%. The distribution of the hydrogen volume fraction in the pipeline under different pressure conditions is shown in Fig 14. Hydrogen accumulation occurred at 8 m, 4 m, and 3 m from the valve at pressures of 1 MPa, 3 MPa, and 5 MPa, respectively. Fig 15 shows the variation in hydrogen volume fraction in the direction along the pipeline. The gradients of hydrogen volume fraction were 0.0118%, 0.0327%, and 0.0533% at 1 MPa, 3 MPa, and 5 MPa, respectively, and the gradient of hydrogen volume fraction at 1 MPa was approximately 1/5 of that at 5 MPa. The actual delivery should be as low as possible to reduce hydrogen accumulation.

**Fig 16:**
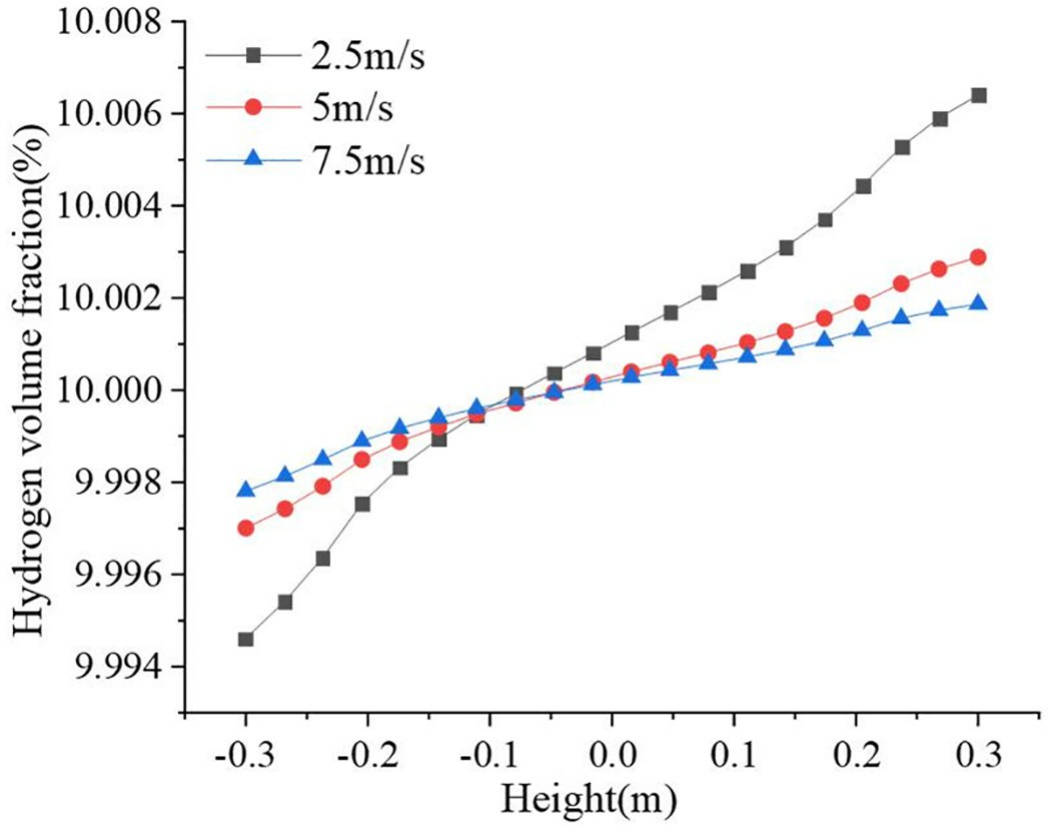
Variation of hydrogen volume fraction at different inlet velocities.

The hydrogen blending ratio was set to 10%, and the inlet velocities were 2.5 m/s, 5 m/s, and 7.5 m/s. Fig 16 shows the variation in hydrogen volume fraction in the pipeline. The hydrogen volume fraction gradients were 0.0059% and 0.0041% at inlet velocities of 5 m/s and 7.5 m/s, respectively. The hydrogen accumulation was more pronounced at a flow rate of 2.5 m/s. The maximum hydrogen volume fraction is 10.0064%, and the gradient reaches 0.0118%, which is more than twice as much as that at 5 m/s and 7.5 m/s flow rates. The higher the velocity at the inlet, the higher the turbulent kinetic energy of the gas and the higher the turbulence intensity. The increased flow velocity enhanced the intensity of the disturbance in the pipeline and promoted further gas blending. Therefore, it is important to increase the velocity of hydrogen-blended natural gas in the transport pipeline as much as possible during transport. Higher velocities can reduce the buildup of hydrogen to some extent, but the problem of energy consumption associated with maintaining higher velocities cannot be ignored. When hydrogen flows at higher velocities in a pipeline, it faces greater fluid resistance. This resistance comes from friction between the hydrogen and the inner walls of the pipe, as well as internal friction between the hydrogen molecules. To overcome these resistances, more energy is expended to propel the hydrogen flow. Hydrogen-blended natural gas undergoes a conversion between kinetic energy and pressure energy during flow, and high velocity flow exacerbates the energy loss during this conversion. In order to maintain a certain pressure level, additional energy needs to be consumed to replenish these losses. Therefore, smooth inner walls and suitable pipeline diameters can be used to reduce frictional resistance, and the selection of efficient, energy-saving equipment (such as pumps, compressors, etc.) can reduce energy consumption.

## 5. Conclusion

During the transportation of hydrogen-blended natural gas, the accumulation of hydrogen can affect the transportation safety of the delivery pipeline. In this paper, the influence of the undulation angle, undulation height, pipeline diameter of the undulation pipeline as well as the ball valve opening, inlet pressure, temperature, velocity, hydrogen blending ratio and other parameters on the distribution of hydrogen concentration in the pipeline are calculated and analyzed by using the CFD method. The specific conclusions are as follows:

1. The greater the rise angle of the undulation line, the stronger the impact of the gas on the right-hand wall, and the less likely it is that accumulation will occur. Increasing the rise angle from 30° to 60° reduces the hydrogen volume fraction gradient by 30%. The higher the height of the ascent, the more pronounced the hydrogen accumulation effect. An increase from 5 m to 15 m increased the hydrogen volume fraction gradient by a factor of 1.3. The change in the gradient was smoother for larger pipeline sizes, which were less prone to accumulation.
2. As the ball valve opening increased, the change in velocity decreased, and hydrogen accumulation became more pronounced. Increasing the opening from 25% to 100% increased the hydrogen volume fraction gradient by more than a factor of two. It is desirable to select low opening conditions for transportation; however, the fluid impact is higher at low openings.
3. When the ball valve opening was constant, the higher the hydrogen blending ratio, the easier it was for hydrogen gas to accumulate. The higher the temperature of the inlet gas, the more violent the movement of the gas molecules in the pipeline. This provides better resistance to hydrogen accumulation owing to gravitational factors. Therefore, low temperatures should be avoided during actual transportation. The higher the pressure, the more pronounced the hydrogen accumulation. At a pressure of 5 MPa, hydrogen accumulated 3 m from the valve. An increase in inlet velocity increases the disturbance in the pipeline, which contributes to blending. Large inlet velocities reduced hydrogen accumulation.
4. Selecting a low hydrogen blending ratio, high temperature, low pressure, and high flow rate transportation conditions can reduce hydrogen accumulation in the transportation pipeline.

The focus should be on the effect of stratification for meter-sized pipelines, gas risers in the teens, and even hydrogen-blended natural gas pipelines with even greater height differences. The larger the pipeline diameter the smoother the change in the hydrogen volume fraction gradient and the less prone to stratification. In the valve pipeline, the low opening makes the flow rate in the ball valve higher, and the shock generated by the high flow rate is easy to damage the ball valve, but promotes the blending of hydrogen-blended natural gas. The higher the gas temperature, the more intense the molecular movement, which can better resist the gas stratification caused by gravity. Therefore, it is easy to choose low opening, high temperature, low hydrogen-blending ratio, low pressure and high flow rate for hydrogen-blended natural gas, which can significantly reduce the probability of hydrogen aggregation and stratification phenomenon, and at the same time, it is easy to install monitoring equipment. In the future, the risk can be reduced from three aspects: structural design, operating conditions, and monitoring equipment to realize the safe delivery of hydrogen-blended natural gas.

## Supporting information

S1 Data (DOCX)
