# Supplementary material for: Simulation study on hydrogen concentration distribution in hydrogen blended natural gas transportation pipeline
Source: PLoS One. 2024 Dec 3;19(12):e0314453. doi: 10.1371/journal.pone.0314453 (PMC11614214; doi:10.1371/journal.pone.0314453)
Supplement: S1 Data — (DOCX) [file pone.0314453.s001.docx]

Supporting Information

Simulation study on hydrogen concentration distribution in hydrogen blended natural gas transportation pipeline

**Weiqing Xu, ^1,2^ Yongwei An, ^3^ Shuangjie Yan, ^3^ Rui Li, ^4^ Maolin Cai, ^1,2^ Guanwei Jia, ^2,3,*^**

^1^ School of Automation Science and Electrical Engineering, Beihang University, Beijing 100191, China

^2^ Pneumatic and Thermodynamic Energy Storage and Supply Beijing Key Laboratory, Beijing 100191, China

^3^ School of Physics and Electronics, Henan University, Kaifeng 475004, China

^4^ General Institute of Science and Technology of National Petroleum and Natural Gas Pipeline Network Group Co.,Ltd, Langfang, 065000, Hebei, China

^*^ Corresponding author: jiaguanwei@henu.edu.cn; Tel./Fax: +86-371-23881602

**Table S1 data in Fig 4**

| x/m | 30° | 45° | 60° |
| --- | --- | --- | --- |
| 0.3 | 10.01833 | 10.01517 | 10.01517 |
| 0.268 | 10.01732 | 10.0136 | 10.01424 |
| 0.237 | 10.01555 | 10.01143 | 10.01259 |
| 0.205 | 10.013 | 10.00883 | 10.01038 |
| 0.174 | 10.01063 | 10.00668 | 10.00836 |
| 0.142 | 10.00841 | 10.00495 | 10.00662 |
| 0.111 | 10.00654 | 10.00362 | 10.00509 |
| 0.0789 | 10.00499 | 10.0027 | 10.0038 |
| 0.0474 | 10.00371 | 10.00208 | 10.00286 |
| 0.0158 | 10.00275 | 10.00168 | 10.00227 |
| -0.0158 | 10.00202 | 10.00144 | 10.00183 |
| -0.0474 | 10.0015 | 10.00133 | 10.00154 |
| -0.0789 | 10.00106 | 10.00128 | 10.0013 |
| -0.111 | 10.00053 | 10.00122 | 10.00111 |
| -0.142 | 9.99972 | 10.00105 | 10.00087 |
| -0.174 | 9.99835 | 10.00056 | 10.00048 |
| -0.205 | 9.99617 | 9.99938 | 9.99964 |
| -0.237 | 9.99249 | 9.99682 | 9.99752 |
| -0.268 | 9.98887 | 9.99385 | 9.99465 |
| -0.3 | 9.98648 | 9.99133 | 9.99265 |

**Table S2 data in Fig 5**

| x/m | 5 m | 10 m | 15 m |
| --- | --- | --- | --- |
| 0.3 | 10.01517 | 10.01531 | 10.01871 |
| 0.268 | 10.0136 | 10.0143 | 10.01765 |
| 0.237 | 10.01143 | 10.01256 | 10.01574 |
| 0.205 | 10.00883 | 10.01012 | 10.01287 |
| 0.174 | 10.00668 | 10.00806 | 10.0103 |
| 0.142 | 10.00495 | 10.00628 | 10.00798 |
| 0.111 | 10.00362 | 10.00485 | 10.00606 |
| 0.0789 | 10.0027 | 10.00377 | 10.00447 |
| 0.0474 | 10.00208 | 10.00295 | 10.00332 |
| 0.0158 | 10.00168 | 10.00246 | 10.00221 |
| -0.0158 | 10.00144 | 10.00209 | 10.00118 |
| -0.0474 | 10.00133 | 10.00177 | 10.00031 |
| -0.0789 | 10.00128 | 10.00142 | 9.99947 |
| -0.111 | 10.00122 | 10.00096 | 9.99854 |
| -0.142 | 10.00105 | 10.00021 | 9.99756 |
| -0.174 | 10.00056 | 9.99902 | 9.9963 |
| -0.205 | 9.99938 | 9.99715 | 9.99468 |
| -0.237 | 9.99682 | 9.99393 | 9.99192 |
| -0.268 | 9.99385 | 9.99068 | 9.98926 |
| -0.3 | 9.99133 | 9.98859 | 9.9875 |

**Table S3 data in Fig 6**

| x/m | 400 mm | 600 mm | 800 mm |
| --- | --- | --- | --- |
| 0.3 | 10.01592 | 10.01517 | 10.01486 |
| 0.268 | 10.01432 | 10.0136 | 10.0139 |
| 0.237 | 10.01259 | 10.01143 | 10.01122 |
| 0.205 | 10.00992 | 10.00883 | 10.00877 |
| 0.174 | 10.00756 | 10.00668 | 10.00686 |
| 0.142 | 10.00577 | 10.00495 | 10.00532 |
| 0.111 | 10.0044 | 10.00362 | 10.00412 |
| 0.0789 | 10.00324 | 10.0027 | 10.00322 |
| 0.0474 | 10.00209 | 10.00208 | 10.0025 |
| 0.0158 | 10.00122 | 10.00168 | 10.002 |
| -0.0158 | 10.00049 | 10.00144 | 10.00176 |
| -0.0474 | 9.99973 | 10.00133 | 10.0016 |
| -0.0789 | 9.99897 | 10.00128 | 10.00157 |
| -0.111 | 9.99793 | 10.00122 | 10.00154 |
| -0.142 | 9.99654 | 10.00105 | 10.00148 |
| -0.174 | 9.99471 | 10.00056 | 10.00122 |
| -0.205 | 9.99229 | 9.99938 | 10.00062 |
| -0.237 | 9.98916 | 9.99682 | 9.99897 |
| -0.268 | 9.98697 | 9.99385 | 9.99528 |
| -0.3 | 9.98502 | 9.99133 | 9.99239 |

**Table S4 data in Fig 10**

| x/m | 25% | 50% | 75% | 100% |
| --- | --- | --- | --- | --- |
| 0.3 | 10.00531 | 10.0064 | 10.00674 | 10.01071 |
| 0.268 | 10.00485 | 10.00591 | 10.0062 | 10.00971 |
| 0.237 | 10.00417 | 10.00528 | 10.00557 | 10.00786 |
| 0.205 | 10.00346 | 10.00443 | 10.00474 | 10.00548 |
| 0.174 | 10.00291 | 10.00371 | 10.00394 | 10.00354 |
| 0.142 | 10.0024 | 10.0031 | 10.00321 | 10.00181 |
| 0.111 | 10.00193 | 10.0026 | 10.00258 | 10.00058 |
| 0.0789 | 10.00156 | 10.00212 | 10.00206 | 9.99982 |
| 0.0474 | 10.00121 | 10.0017 | 10.00155 | 9.99968 |
| 0.0158 | 10.0009 | 10.00126 | 10.00106 | 9.99995 |
| -0.0158 | 10.00059 | 10.00081 | 10.0006 | 10.00024 |
| -0.0474 | 10.00029 | 10.00038 | 10.00016 | 10.00053 |
| -0.0789 | 9.99992 | 9.99993 | 9.99972 | 10.00069 |
| -0.111 | 9.99952 | 9.99947 | 9.99921 | 10.00077 |
| -0.142 | 9.9991 | 9.99894 | 9.99864 | 10.00069 |
| -0.174 | 9.9986 | 9.99833 | 9.99793 | 9.99967 |
| -0.205 | 9.99802 | 9.99754 | 9.99699 | 9.99783 |
| -0.237 | 9.99697 | 9.99637 | 9.99595 | 9.99468 |
| -0.268 | 9.99616 | 9.99542 | 9.99503 | 9.99145 |
| -0.3 | 9.99553 | 9.99461 | 9.99413 | 9.9894 |

**Table S5 data in Fig 12**

| x/m | 10% | 20% | 30% |
| --- | --- | --- | --- |
| 0.3 | 0.0064 | 0.01022 | 0.01174 |
| 0.268 | 0.00591 | 0.00944 | 0.01084 |
| 0.237 | 0.00528 | 0.00845 | 0.0097 |
| 0.205 | 0.00443 | 0.0071 | 0.00815 |
| 0.174 | 0.00371 | 0.00595 | 0.00683 |
| 0.142 | 0.0031 | 0.00498 | 0.00571 |
| 0.111 | 0.0026 | 0.00417 | 0.00479 |
| 0.0789 | 0.00212 | 0.00341 | 0.00392 |
| 0.0474 | 0.0017 | 0.00272 | 0.00313 |
| 0.0158 | 0.00126 | 0.00203 | 0.00233 |
| -0.0158 | 8.12E-04 | 0.00131 | 0.00152 |
| -0.0474 | 3.78E-04 | 6.17E-04 | 7.25E-04 |
| -0.0789 | -6.76E-05 | -9.36E-05 | -8.52E-05 |
| -0.111 | -5.25E-04 | -8.21E-04 | -9.14E-04 |
| -0.142 | -0.00106 | -0.00167 | -0.00188 |
| -0.174 | -0.00167 | -0.00264 | -0.003 |
| -0.205 | -0.00246 | -0.00388 | -0.00442 |
| -0.237 | -0.00363 | -0.00574 | -0.00653 |
| -0.268 | -0.00458 | -0.00724 | -0.00825 |
| -0.3 | -0.00539 | -0.00852 | -0.00972 |

**Table S6 data in Fig 13**

| x/m | 273 K | 298 K | 323 K |
| --- | --- | --- | --- |
| 0.3 | 10.0064 | 10.00579 | 10.00545 |
| 0.268 | 10.00591 | 10.00533 | 10.00503 |
| 0.237 | 10.00528 | 10.00476 | 10.0045 |
| 0.205 | 10.00443 | 10.00398 | 10.00378 |
| 0.174 | 10.00371 | 10.00332 | 10.00316 |
| 0.142 | 10.0031 | 10.00276 | 10.00264 |
| 0.111 | 10.0026 | 10.0023 | 10.00221 |
| 0.0789 | 10.00212 | 10.00187 | 10.00181 |
| 0.0474 | 10.0017 | 10.00148 | 10.00144 |
| 0.0158 | 10.00126 | 10.00108 | 10.00107 |
| -0.0158 | 10.00081 | 10.00067 | 10.00069 |
| -0.0474 | 10.00038 | 10.00028 | 10.00032 |
| -0.0789 | 9.99993 | 9.99987 | 9.99994 |
| -0.111 | 9.99947 | 9.99946 | 9.99955 |
| -0.142 | 9.99894 | 9.99897 | 9.9991 |
| -0.174 | 9.99833 | 9.99841 | 9.99858 |
| -0.205 | 9.99754 | 9.9977 | 9.99792 |
| -0.237 | 9.99637 | 9.99663 | 9.99693 |
| -0.268 | 9.99542 | 9.99576 | 9.99613 |
| -0.3 | 9.99461 | 9.99502 | 9.99545 |

**Table S7 data in Fig 15**

| x/m | 1 MPa | 3 MPa | 5 MPa |
| --- | --- | --- | --- |
| 0.3 | 10.0064 | 10.01746 | 10.02881 |
| 0.268 | 10.00591 | 10.01607 | 10.02652 |
| 0.237 | 10.00528 | 10.01431 | 10.02365 |
| 0.205 | 10.00443 | 10.01193 | 10.01978 |
| 0.174 | 10.00371 | 10.00992 | 10.01653 |
| 0.142 | 10.0031 | 10.00824 | 10.01379 |
| 0.111 | 10.0026 | 10.00684 | 10.01153 |
| 0.0789 | 10.00212 | 10.00553 | 10.00941 |
| 0.0474 | 10.0017 | 10.00435 | 10.00749 |
| 0.0158 | 10.00126 | 10.00316 | 10.00553 |
| -0.0158 | 10.00081 | 10.00193 | 10.00351 |
| -0.0474 | 10.00038 | 10.00074 | 10.00156 |
| -0.0789 | 9.99993 | 9.99952 | 9.99956 |
| -0.111 | 9.99947 | 9.99827 | 9.9975 |
| -0.142 | 9.99894 | 9.9968 | 9.99511 |
| -0.174 | 9.99833 | 9.9951 | 9.99236 |
| -0.205 | 9.99754 | 9.99294 | 9.98885 |
| -0.237 | 9.99637 | 9.98968 | 9.98352 |
| -0.268 | 9.99542 | 9.98703 | 9.97919 |
| -0.3 | 9.99461 | 9.98475 | 9.97549 |

**Table S8 data in Fig 16**

| x/m | 2.5 m/s | 5 m/s | 7.5 m/s |
| --- | --- | --- | --- |
| 0.3 | 10.0064 | 10.00288 | 10.00187 |
| 0.268 | 10.00591 | 10.00263 | 10.00173 |
| 0.237 | 10.00528 | 10.00231 | 10.00156 |
| 0.205 | 10.00443 | 10.0019 | 10.0013 |
| 0.174 | 10.00371 | 10.00156 | 10.00107 |
| 0.142 | 10.0031 | 10.00127 | 10.00088 |
| 0.111 | 10.0026 | 10.00104 | 10.00072 |
| 0.0789 | 10.00212 | 10.00081 | 10.00057 |
| 0.0474 | 10.0017 | 10.00061 | 10.00043 |
| 0.0158 | 10.00126 | 10.0004 | 10.00028 |
| -0.0158 | 10.00081 | 10.00018 | 10.00012 |
| -0.0474 | 10.00038 | 9.99995 | 9.99995 |
| -0.0789 | 9.99993 | 9.99972 | 9.99978 |
| -0.111 | 9.99947 | 9.99948 | 9.99961 |
| -0.142 | 9.99894 | 9.9992 | 9.9994 |
| -0.174 | 9.99833 | 9.99888 | 9.99917 |
| -0.205 | 9.99754 | 9.9985 | 9.9989 |
| -0.237 | 9.99637 | 9.99791 | 9.99849 |
| -0.268 | 9.99542 | 9.99743 | 9.99813 |
| -0.3 | 9.99461 | 9.99701 | 9.99781 |
